# Down-regulated C4orf19 confers poor prognosis in colon adenocarcinoma identified by gene co-expression network

**DOI:** 10.7150/jca.63635

**Published:** 2022-01-09

**Authors:** Wanpeng Wang, Xiaolu Lin, Ran Yu, Suqin Zhou, Yanyan Liu, Haifeng Jia, Feng Han, Yanzhi Bu, Juan Pu

**Affiliations:** 1 Department of Radiotherapy, Lianshui County People's Hospital, Kangda college of Nanjing Medical University, Huai'an city, Jiangsu Province 223400, China.; 2 Department of Central Laboratory, Lianshui County People's Hospital, Kangda college of Nanjing Medical University, Huai'an city, Jiangsu Province 223400, China.; 3 Department of Digestive Endoscopy, Fujian Provincial Hospital, Provincial Clinic Medical College, Fujian Medical University, Fuzhou city, Fujian Province 350001, China.; 4 Department of General Surgery, Lianshui County People's Hospital, Kangda college of Nanjing Medical University, Huai'an city, Jiangsu Province 223400, China.

**Keywords:** Colorectal cancer (CRC), Colon adenocarcinoma (COAD), weighted gene co-expression network analysis (WGCNA), C4orf19, Prognosis

## Abstract

Colon adenocarcinoma (COAD) is the most common histologic subtype of colorectal cancer (CRC), and its prognosis is poor. Unlike traditional research in molecular biology, which is limited to analyzing the function of a single gene or protein in malignant tumors. The Weighted gene correlation network analysis (WGCNA) technique is used to describe the gene association model among different samples in order to identify highly collaborative genes. In this study, a computational strategy was used to conduct a systematic study of prognosis-related genes (PRGs) of COAD from the TCGA database. PRGs were subsequently used for WGCNA, which included 379 COAD patient expression profiles and 39 controls. As a consequence, nine gene modules were built. Among these, the brown module had not only a negative relationship with COAD, but also simultaneously in inverse relationship with the clinical stage, stage T, stage M and stage N. C4orf19, which was identified as one of the DEG and hub genes in the brown module by calculating modular connectivity, has a negative correlation with the clinical stage and TMN stage. In addition, the downward-regulated C4orf19 protein was detected in COAD clinical specimens. Finally, *in vitro* experiments have confirmed that regulated C4orf19 can promote COAD cell proliferation, invasion and migration, and the biological mechanism of C4orf19 perhaps by influencing the nitrogen metabolic pathway.

## Introduction

Colorectal cancer (CRC) has become the third most commonly occurring cancer in the world after breast and lung cancer 1. In recent years, the incidence of CRC and the number of deaths has been increasing, while the five-year survival rate is still low 2. CRC comprises predominantly colon and rectal cancer, and adenocarcinoma of the colon (COAD) has the highest incidence. The pathogenesis of COAD is still not clear, while it is generally believed that the pathogenesis of COAD is related to a variety of factors, such as genetics and poor dietary habits 3. The onset of COAD is insidious, and it is easy to be ignored by patients because there is only mild abdominal pain, diarrhea, and stool occult blood in the early stage of the disease. With the development of COAD, tumor cells enter the blood and lymph leading to the emergence of metastases, making the treatment of colorectal cancer unsuccessful. As a result, the early diagnosis of colorectal cancer is very important to its treatment.

In the development of microarray technology and gene chip technology, genomics, transcriptomics, proteomics, and metabonomics have been used to reveal the changes of genes, proteins and metabolism in malignant tumors 4, 5. Different from the traditional molecular biology research, which is limited to the analysis of the function of a single gene or protein in malignant tumors, omics technology can systematically analyze the expression differences of a large number of genes or proteins, enabling scholars to conduct comprehensive research on tumors 6. The Weighted Gene Correlation Network Analysis (WGCNA) technique is used to describe the gene association model between various samples to identify highly collaborative gene sets 7-9. At the same time, the weighted method excludes false negative or false positive results, so that the module specificity after analysis is stronger, and the obtained hub genes have a higher correlation with tumors. In this study, we used a computational strategy to conduct a systematic study of prognostic genes (RPGs) from the COAD database. RPGs were subsequently used for the WGCNA, which included 379 COAD expression profiles and 39 controls. The WGCNA results combined with DAVID (Database for Annotation, Visualization and Integrated Discovery) were used to identify the specific module associated with the COAD pathology parameters and the hub genes were detected by intramodular connectivity. Finally, the siRNA of the hub gene was used to validate the role of the hub gene in the physiological function of COAD cell lines.

## Materials and methods

### Dataset collection and human COAD tissue samples

The COAD RNA-Seq gene expression profiles and clinical information were downloaded from the TCGA database (https://portal.gdc.cancer.gov/) to the COAD project 10. The raw expression data were collected by the Perl programming language (5.32.0)^11^, ^12^. The transformation from the official gene symbol to the identification (ID) was performed using the programming language R and Perl. In addition, the R programming language was used to extract and consolidate clinical information, including survival time, survival status, age, gender, clinical stage and TNM stage. Consequently, 379 COAD patients with comprehensive survival information and 39 normal tissues were screened for further analysis. The differentially expressed genes (DEGs) were screened by use of the Linear Models for Microarray Data (LIMMA) package in Bioconductor, based on a cutoff criteria of adjusting *P* Value (FDR) < 0.05 and |fold change (FC) | > 2, adjusted method used the Benjamini & Hochberg 13. Forty COAD patients, together with adjacent normal tissue samples, were collected from Lianshui County People's Hospital. Inclusion criteria: (1). ESCC was diagnosed by the department of pathology; (2). The patients did not receive chemoradiotherapy before sampling. (3). The patients had no history of infection or hematologic disease in the past three months. Exclusion criteria: (1). The clinical data of patients were incomplete; (1). The follow-up of patients was missing; (3). The patients received blood transfusion, recently; (4). The patients with severe infections or autoimmune diseases. All patients had given their written consent, which was done in accordance with the Helsinki Declaration and approved by the Lianshui County People's Hospital Ethics Board.

### Functional enrichment analysis

DAVID (Database for Annotation, Visualization and Integrated Discovery) (http://david.abcc.ncifcrf.gov/) 14 was used to analysis the functions or pathways of the DEGs or genes in special modules, which is a gene functional classification tool to integrate a set of functional annotation and analyze biological functions behind massive genes. KEGG (Kyoto Encyclopedia of Genes and Genomes, http://www.genome.jp/) provides a knowledge base for the systematic analysis of gene functions, linking genomic information to higher-order functional information 15. The current study considers FDR < 0.05 as a statistically significant difference. For DEG enrichment, the DEG was divided into an up-regulated group and a downregulated group, and the gene ontology (GO) analysis was performed respectively. The top 20 biological process (BP) was visualized using the R package ggplot2 16.

### Overall survival rate analysis

A total of 379 COAD patients with comprehensive prognosis data were used for prognosis analysis. Based on the median of the expression value, patients were divided into one low expression group and one high expression group. Kaplan-Meier analysis was used to generate the *P*-value of the logistics rank. As a result, 3170 genes with a *P* value below 0.05 were selected for construction of the co-expression network. The flowchart for obtaining the gene co-expression network and experimental validation was drawn as shown in Supplementary Figure. S1.

### Construction of prognosis related-genes co-expression network

To explore the between prognosis related genes of COAD and construct gene co-expression network construction, the “WGCNA” R package was used to convert co-expression measures into connections weight or topology overlap measure 17. The co-expression method was commonly used to explore correlations in gene expression. Genes involved in the same pathway or functional compound tend to exhibit a similar expression pattern 18. Thus, the development of a genetic co-expression network facilitates the identification of genes with similar biological functions 19. In this study, 3170 genes whose log-rank *P* value was less than 0.05 and 379 COAD samples containing clinical information and 39 normal samples were entered to construct weighted co-expression modules. The Pearson correlation matrix was converted to the weighted adjacency matrix by use of the formula amn = |cmn|^β^(cmn represents the Pearson correlation between genes, amn represents the adjacency between genes, β parameter can amplify the correlation between genes). The soft threshold power β was determined based on the standard scale-free network 20. Afterwards, we converted the adjacency relationship into a topological overlapping matrix (TOM) and hierarchical genes to identify modules containing similar genes. The settings have been configured as follows: TOMType = "unsigned", minModuleSize = 30, reassignThreshold = 0, mergeCutHeight = 0.25, numericLabels = TRUE, pamRespectsDendro = FALSE.

### Gene Set Enrichment Analysis (GSEA)

A total of 379 COAD samples in TCGA were divided into high and low expression groups based on the median expression levels of the mediated gene. To study the potential mechanisms of hub genes in COAD, GSEA between the two groups was performed using the Java GSEA implementation 21, where FDR < 0.05 was set as the cut-off criteria.

### Cell culture and siRNA interference

HT-8, HT-29, SW480, Caco2 and HCT-116 human cell lines were obtained from the American Type Culture Collection (ATCC) and cultured in the medium recommended by the ATCC with 10% fetal bovine serum (Beyotime Biotechnology, China). Both C4orf19 siRNA and Scramble siRNA control were acquired from Thermo Fisher Scientific Inc. Transfection was performed with Lipofectamine 2000 reagent (Thermo Fisher Scientific, USA), according to the manufacturer's protocol. Forty-eight hours after transfection, the proteins were collected. All experiments were performed in triplicate.

### Protein analysis of the cell line

Western blotting was used for analysis for proteins of C4orf19, Carbonic anhydrase II (CA2) and Carbonic anhydrase IV (CA4). In brief, the cells were assayed with RIPA buffer (Thermo Fisher Scientific, USA) respectively, and western blotting was performed using standard procedures. The primary antibodies used for the analysis were C4orf19 goat polyclonal antibody (Thermo Fisher Scientific, USA), CA2 and CA4 rabbit polyclonal antibody (Proteintech Group, China), and anti-rabbit horseradish peroxidase-conjugated (HRP) antibodies (Proteintech, China) were used as secondary antibodies. Immunolabelled proteins were detected with increased chemiluminescence (Beyotime Biotechnology, China).

### CCK8 assay

Cell proliferation capability was reviewed by Cell Counting Kit-8 (CCK8, Beyotime). Following transfection, the cells were pressed into 96-well slabs and cultured for 24, 48, 72 and 96 hours. 10% CCK8 was added to each well for 4 h at 37℃. The absorbance value was detected at a wavelength of 450 nm by a microplate scanner.

### Wound healing assay

NC siRNA and C4orf19 siRNA were transfected into Caco2 cells, respectively. Then, the transfected cells were seeded into 6-well plates at a density of 3×10^5^ cells/well. When cells attained confluent monolayers, a line, in the center of each well, was drawn using a 1ml pipette tip for producing wound area. Cells were washed three times with phosphate-buffered saline (PBS) to remove the non-adherent cells. The wound healing at 0h and 24h was recorded and the images were photographed at a magnification of ×100.

### Cell invasion assays

NC siRNA and C4orf19 siRNA were transfected into Caco2 cells, respectively. Then, 100μl FBS-free DMEM medium containing 3×10^5^ cells were added to the upper layer. 600μl FBS was added to the lower layer. After 24h, unmigrated cells were removed, and the polycarboester membrane was fixed with methanol. 10 min later of crystal violet staining, cell migration was recorded under the microscope.

### Statistical analysis

Statistical Program for Social Sciences (SPSS) 20.0 software (SPSS Inc., Chicago, IL, USA) and R 4.0.3 software (https://www.r-project.org/) were used to perform statistical analysis and to generate figures. Mann-Whitney U tests were performed between two group comparisons of mRNA expression; Kruskal Wallis test was used for grade data. *P* < 0.05 was considered statistically significant.

## Results

### Identification of DEGs in COAD Tissue Samples

**Figure 1:**
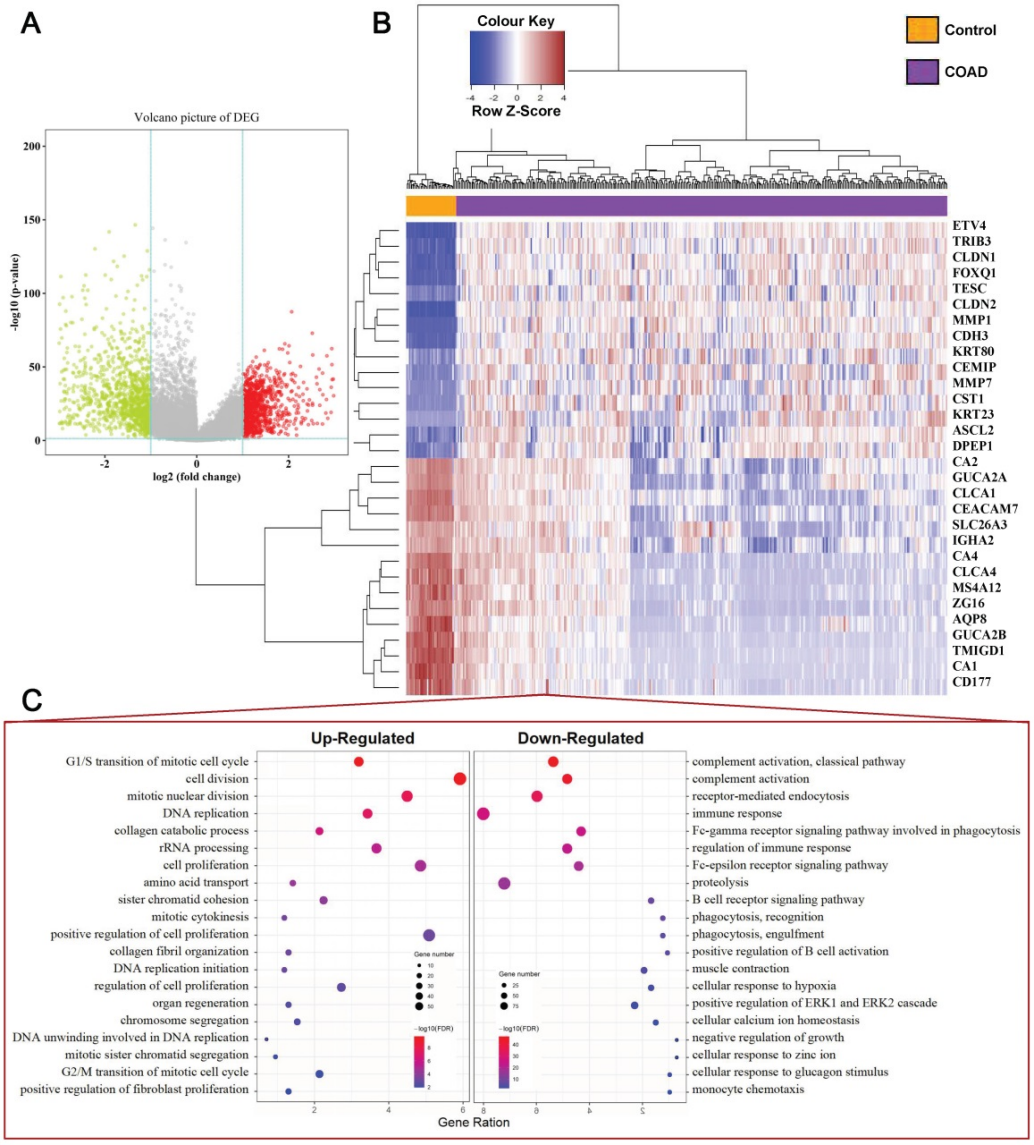
Identification of DEGs in COAD Tissue Samples. (A) Volcano plot of DEGs with |fold change (FC)| > 2 and p-value of <0.05. Red nodes represent DEGs with FC > 2 and p-value of < 0.05; green nodes represent DEGs with FC < 2 and p-value of < 0.05. (B) A heat map of top 15 upregulated and top 15 downregulated DEGs. Each column represents a sample and each row represents one gene. The gradual color ranging from blue to red represents the gene expression changing from downregulation to upregulation. (C) The bubble plot shows the top 20 enriched biological processes (BP) of the upregulated genes and downregulated genes.

Our study concluded that 39 normal tissues and 379 COAD patients and their mRNA expression patterns were present in the TCGA dataset. Based on criteria of adjusting *P* Value < 0.05 and |Fold Change (FC) | > 2, a total of 2119 DEGs (919 upregulated and 1200 downregulated) was identified from the expression profile datasets. The map of volcanoes depicts differential genes **(Figure 1A, Supplementary Table S1)**, whereas the heat map shows top 15 up-regulated and top 15 down-regulated DEGs between normal versus cancer tissues **(Figure 1B)**. Functional enrichment analysis showed that the up-regulated DEGs were significantly enriched in cell proliferation and cell cycle terms, including G1/S transition of mitotic cell cycle, cell division, cell proliferation, DNA replication, G2/M transition of mitotic cell cycle. The downregulated DEGs were associated with immune response terms, such as complement activation, immune response, and positive regulation of B cell activation, etc **(Figure 1C),** and full terms were displayed at **Supplementary Table S2 and S3**.

### Construction of the prognosis-associated genes co-expression network

**Figure 2:**
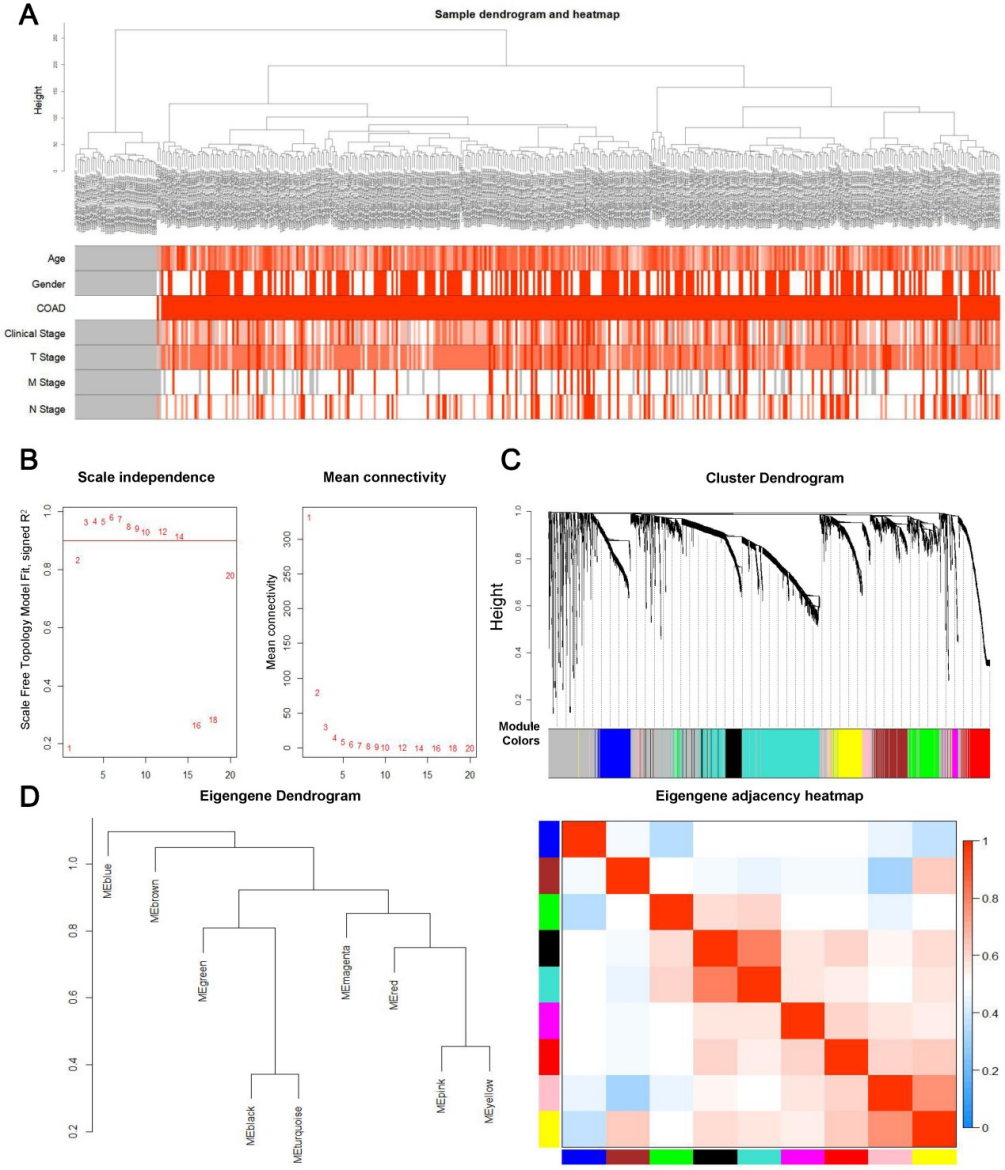
Construction of the prognosis-associated genes co-expression network. (A) Clinical information from samples involved in constructing the gene co-expression network associated with the prognosis. (B) The left panel shows the scale-free fit index, signed Rˆ 2 (y-axis) and the soft threshold power (x-axis). β = 3 has been chosen for subsequent analysis. The right panel shows the mean connectivity (y-axis), which is a strictly decreasing function of the power β (x-axis). (C) Clustering dendrogram of genes. The color bands provide a simple visual comparison of module assignments based on the dynamic tree cutting method. (D) Clustering dendrogram (left) and heatmap of the correlation between module eigengenes (right).

First, overall survival and its significance were calculated by Kaplan-Meier survival analysis and log ranking test. The log-rank *P* value was subsequently calculated. Consequently, 3170 genes with a P-value below 0.05 were selected for WGCNA. A total of 3,170 genes and 418 samples, including 39 controls and 379 tumor samples with partial clinical data, were used in the construction of the prognostic gene co-expression network. Interestingly, the analysis of hierarchical clusters of data revealed that genes associated with prognosis have the value of distinguishing most COAD samples from controls, as shown in the **Figure 2A**, and none of the abnormal samples were found. Then an appropriate flexible threshold was calculated to complete the construction of a free-scale network. After testing powers from 1-20, a power value of 3 was selected, as at this value the connectivity between genes in the network was consistent with a scale-free network distribution, where the curve first reached Rˆ2 > 0.9 **(Figure 2B)**. The co-expression modules were realized through hierarchical clustering and dynamic branch decoupling, leading to the generation of 9 modules **(Figure 2C)**. These modules were designated using different colors, with 749, 231, 228, 222, 210, 192, 158, 116 and 48 genes in the turquoise, blue, brown, yellow, green, red, black, pink and magenta modules, respectively. In addition, 1016 genes that did not belong to any module were incorporated into the grey module and were not considered in downstream analyses **(Figure 2C)**. To explore the similarity of co-expression of modules, the eigengenes were calculated and pooled on the basis of correlations (**Figure 2D**). Most modules were not in close proximity to other modules, which meant that the grouping was independent and precise 22.

### Identifier of the COAD modules and functional annotation

**Figure 3:**
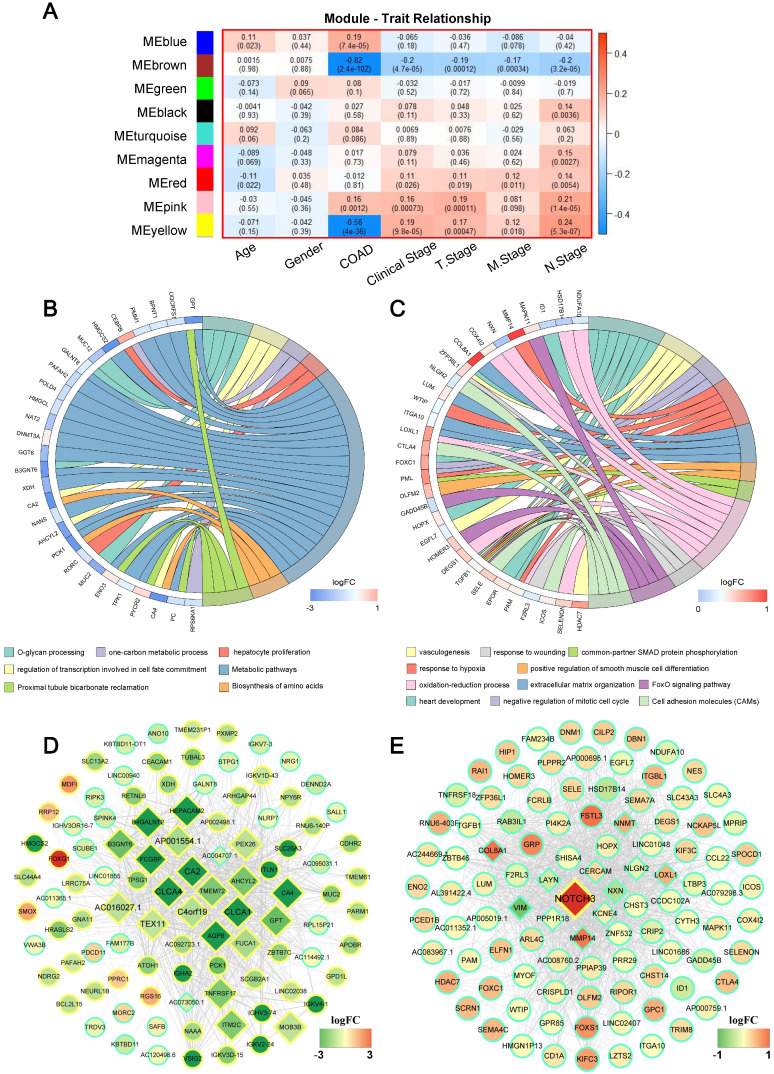
Weighted gene correlation network analysis (WGCNA) identifies critical modules correlating with pathological parameters. (A) Correlation between modules and traits. The upper number in each cell refers to the correlation efficient of each module in the trait, and the lower number is the corresponding *P* value. Gene Ontology (GO) biological process (BP) terms in the enrichment analysis of genes in the turquoise (B) and brown module (C). (D) The hub genes of brown module with weights (w) above a threshold of 0.01. (E) The hub genes of pink module with weights (w) above a threshold of 0.05.

The primary objective of WGCNA is to explore the relationship between gene modules and clinical information, and to identify the modules most relevant to clinical features, which are of significant biological importance. We then detected the correlation between the various modules and the clinical parameters by calculating the correlation coefficient of the eigengens of each module. As displayed in **Figure 3A**, several modules showed significantly associated with certain clinic parameters, for example: the blue (r = 0.19, *P* = 7.4×10^-5^) and pink (r = 0.16, *P* = 0.001) module, showed a very high positive correlation with COAD respectively; While significantly negative correlations were detected between the brown (r = -0.82, *P* = 2.4×10^-102^), yellow (r = -0.56, *P* = 4.0×10^-36^) module with COAD; Meanwhile, the yellow module was associated with clinical stage (r = 0.19, *P* = 9.8×10^-5^), T stage (r = 0.17, *P* = 0.0004), M stage (r = 0.12, *P* = 0.018), N stage (r = 0.24, *P* = 5.3×10^-7^). Interestingly, we found that the blown module not only had a negative relation with COAD, but also simultaneously inversely related to clinical stage (r = -0.20, *P* = 4.7×10^-5^), T stage (r = -0.19, *P* = 0.0001), M stage (r = -0.17, *P* = 0.0003) and N stage (r = -0.20, *P* = 3.2×10^-5^); On the contrary, the pink module was positively significantly correlated with clinical stage (r = 0.16, *P* = 0.00073), T stage (r = 0.19, *P* = 0.00011) and N stage (r = 0.21, *P* = 1.4E-05). Complete module information and clinical parameter relationships have been provided in Supplementary Table S4.

Since brown and pink module were significantly associated with almost all clinical parameters, to further understand the biological functions and pathways of the genes in these two modules, enrichment analyses of GO and KEGG were subsequently performed. **Figure 3B and C** displayed significant BP terms and pathways for brown and pink module respectively. The brown module showed functional enrichment in O-glycan processing, one-carbon metabolic process, hepatocyte proliferation and regulation of transcription involved in cell fate commitment (**Figure 3B**). Pathway analysis was enriched in metabolic pathways, proximal tubule bicarbonate reclamation and Biosynthesis of amino acids (all FDR < 0.05). For pink module, genes in the module were significantly enriched in heart development, negative regulation of mitotic cell cycle, response to hypoxia and so on, and significant KEGG pathways enriched in pink module included a FoxO signaling pathway, Cell adhesion molecules (CAMs) (all FDR < 0.05) **(Figure 3C)**.

### Analysis of hub genes

It is widely acknowledged that highly connected hub nodes are at the heart of the network architecture 23 and some more centralised genes in the network are more likely to be the primary drivers of appropriate cellular function than peripheral genes. 24. For this reason, more important nodes can be identified through identifying hub nodes. In this study, the top 10% of nodes with the highest intra-modular connectivity were defined as hub genes within each module. 25. **Figure 3D** and **Figure 3E** were the weighted co-expression networks of genes in brown and pink module, which only displayed connections with weight (w) above a threshold of 0.01 for brown and 0.05 for pink for the best view. It can be seen that the trend in relative expression for most concentrator genes was consistent and may have high connectivity to neighbouring genes whose functions were consistent with GO and KEGG analytical results **(Figure 3B and E, diamond nodes)**.

**Figure 4:**
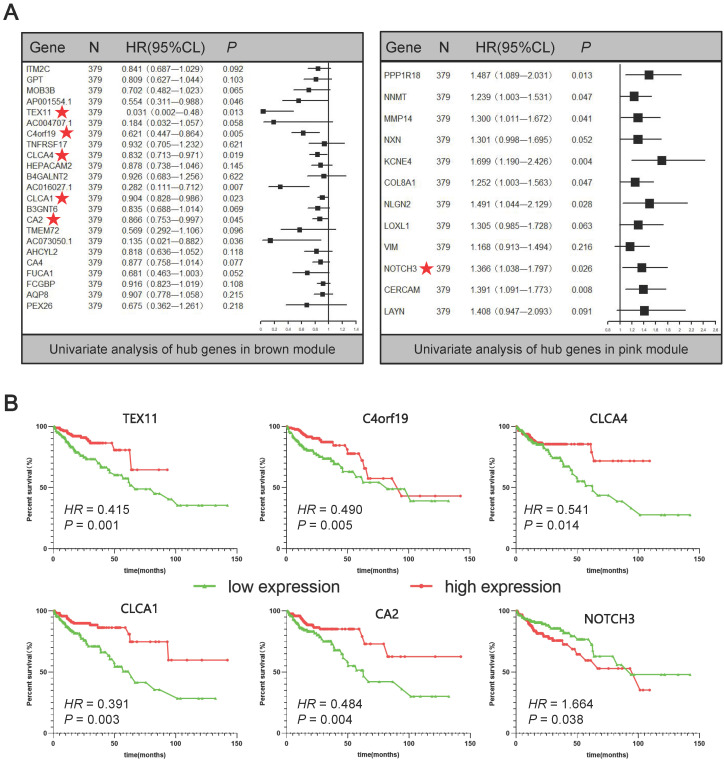
Univariate Cox analyses and Kaplan-Meier curves of the candidate genes. (A) Univariate Cox analyses of hub genes in brown and pink modules. (B) Kaplan-Meier curves of the effect of the gene expression level of the risk genes (TEX11, C4orf19, CLCA4, CLCA1, CA2, NOTCH3) on the prognosis of COAD cancer patients.

In order to further screen for suitable biomarkers, univariate Cox analyses were performed in the brown and pink module hub genes. Results have been presented on **Figure 4A.** On the one hand, a total of 13 prognosis-related genes with both *P*_Cox_ < 0.05 were identified, including 8 protective (Hazard ratio < 1) and 5 hazardous (Hazard ratio > 1) genes. On the other hand, among them, 6 protein-coding genes were defined as DEGs through the above analysis (**Figure 1, Supplementary Figure S2**): TEX11, C4orf19, CLCA4, CLCA1, CA2, NOTCH3 (**Figure 4A**★). Based on this analysis, the six candidate genes were selected for more in-depth analysis. Using the median as the threshold, their Kaplan-Meier curves were plotted as in the **Figure 4B**, expression patterns of 6 biomarker genes were significantly associated with overall survival of COAD patients on the TCGA dataset.

### Relationship between candidate gene expression and clinical outcomes of COAD patients

**Figure 5:**
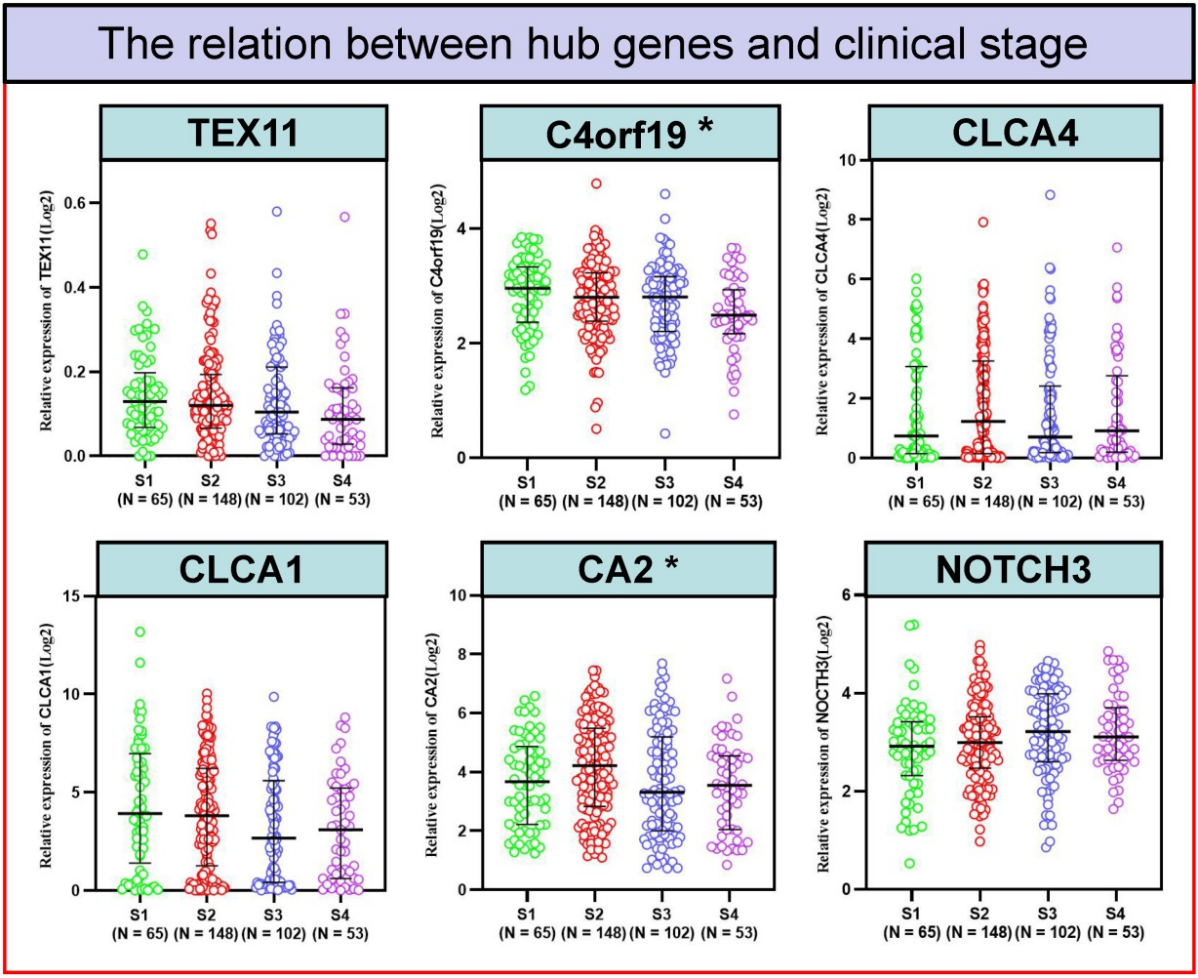
The relationship between hub genes and clinical stage. * *P* < 0.05.

**Figure 6:**
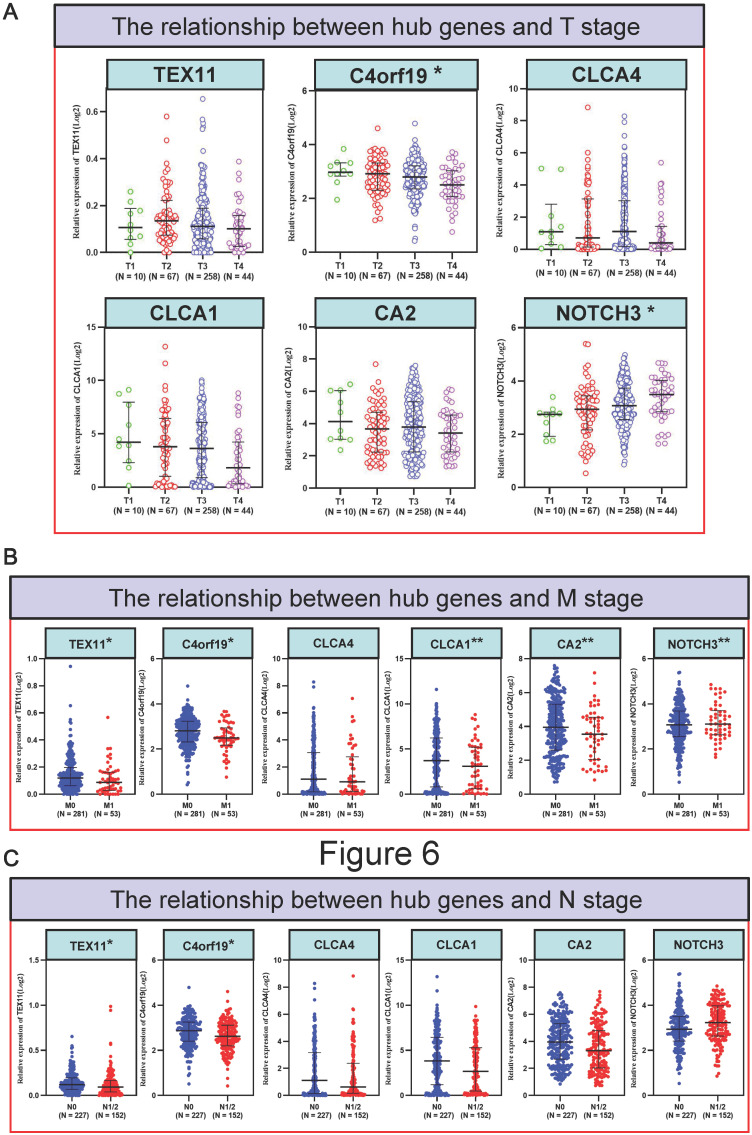
The relationship between hub genes and clinical information of patients with COAD. (A) The relationship between hub genes and T stage; (B) The relationship between hub genes and N stage; (C) The relationship between hub genes and M stage. * *P* < 0.05; ** *P* < 0.01.

To better detect the relation of COAD to these 6 candidate genes in patients. We examined the correlation between the expression of 6 candidate genes and the clinico-pathologic characteristics of COAD patients in the TCGA cohort. Typically, the expression level of C4orf19 (*P* = 0.024) and CA2 (*P* = 0.024) was significantly correlated with the clinical stage of COAD patients **(Figure 5)**. Similarly, the expression level of C4orf19 and CLCA1 was significantly correlated with Stage T in COAD patients **(Figure 6A)**. The tendency to express these candidate genes corresponds to each ME involved in different modules. In addition, expression levels for TEX11, C4orf19, CLCA1 and CA2 were low-regulated in COAD patients with lymph node metastases, while NOTCH 3 was up-regulated in these patients **(Figure 6B).** In addition, both TEX11 and C4orf19 expression levels were low-regulated in COAD patients with distant metastases **(Figure 6C)**. All results suggested that C4orf19 expression levels can be used not only as a biomarker for colorectal cancer occurrence, but also for COAD progression.

### C4ORF19 potentially affects COAD by nitrogen metabolic pathway

**Figure 7:**
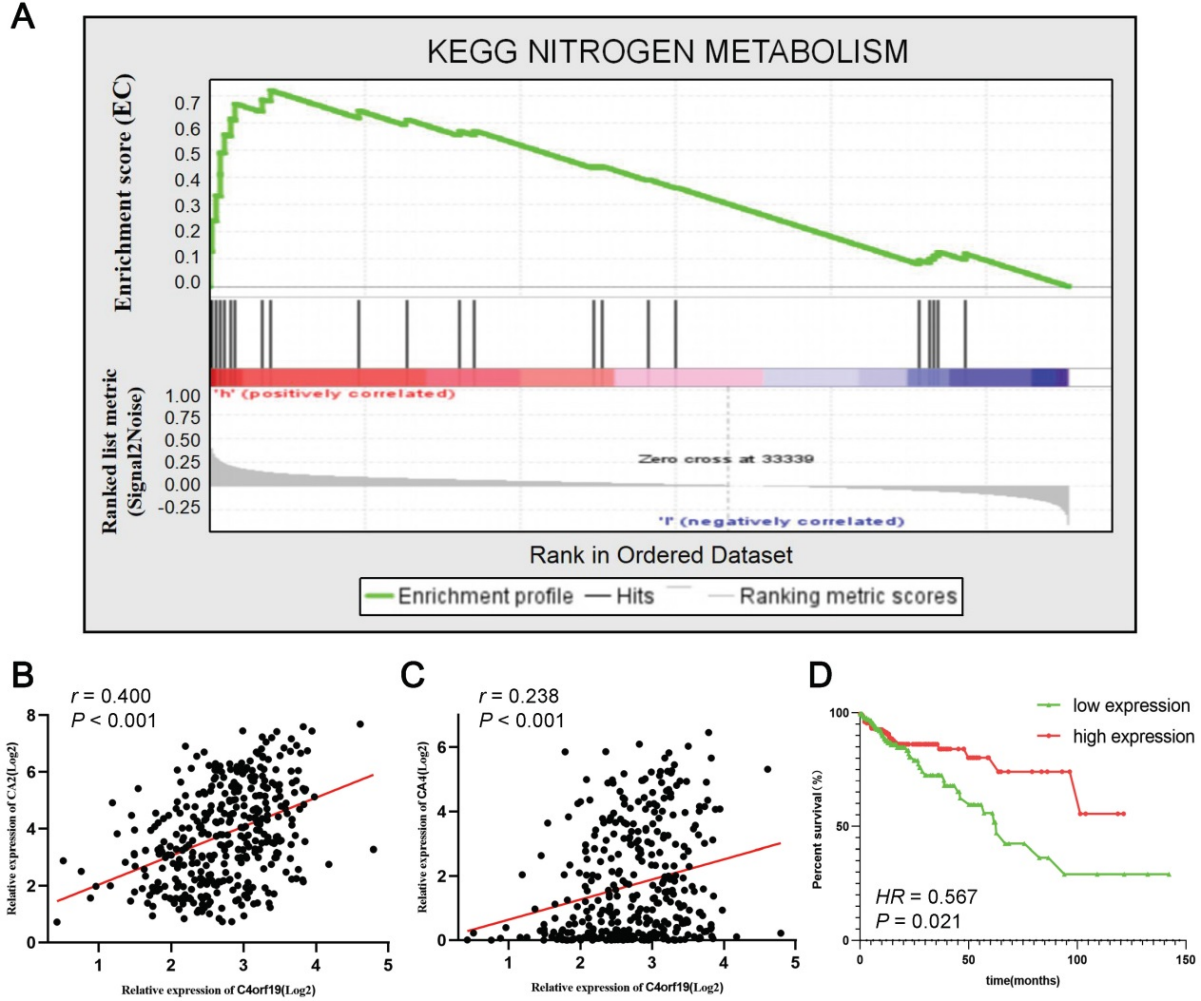
Gene set enrichment analysis of C4orf19 in COAD datasets. (A) GSEA analysis high C4orf16 expression datasets enriched in nitrogen metabolism pathway. (B) The relationship between mRNA expression of C4orf19 and CA2; (C) Relationship between mRNA expression of C4orf19 and CA4; (D) Kaplan-Meier curves of the effect of the gene expression level of the risk genes CA4 on the prognosis of COAD cancer patients.

To identify the signaling pathways that potential C4orf19 biological functions have activated in the COAD, the GSEA analysis was conducted on high and low C4orf16 expression datasets, and the most enriched signaling pathways were selected based on the normalized enrichment score (NES). According to the cut-off criteria, 18 signaling pathways were identified enriched in COAD samples with strongly expressed C4orf16, as shown in** Supplementary Table S5** and **Supplementary Figure. S3**. The priority pathway for these was the nitrogen metabolic pathway with the highest NES **(Figure 7A)**. It should be noted that there has been considerable research on the role of the nitrogen metabolic pathway in COAD 26, 27, and has received growing attention over the last few years. It is interesting to note that the 10 genes were identified as core enrichment genes with the highest metric score in the nitrogen metabolic pathway in this analysis **(Supplementary Figure S4)**, and among them CA2 and CA4 as well as C4orf19 was nominated for the brown module in WGCNA. Positive correlations were identified between the expression of the mRNA of C4orf19 and CA2** (Figure 7B)** or CA4 **(Figure 7C),** and the expression of CA4 also has value in judging the prognosis of COAD patients** (Figure 7D)**. All these results indicated that the loss of expression of C4orf19 may cause the progression of COAD by influencing the nitrogen metabolic pathway.

### C4orf19 expression and role in COAD

**Figure 8:**
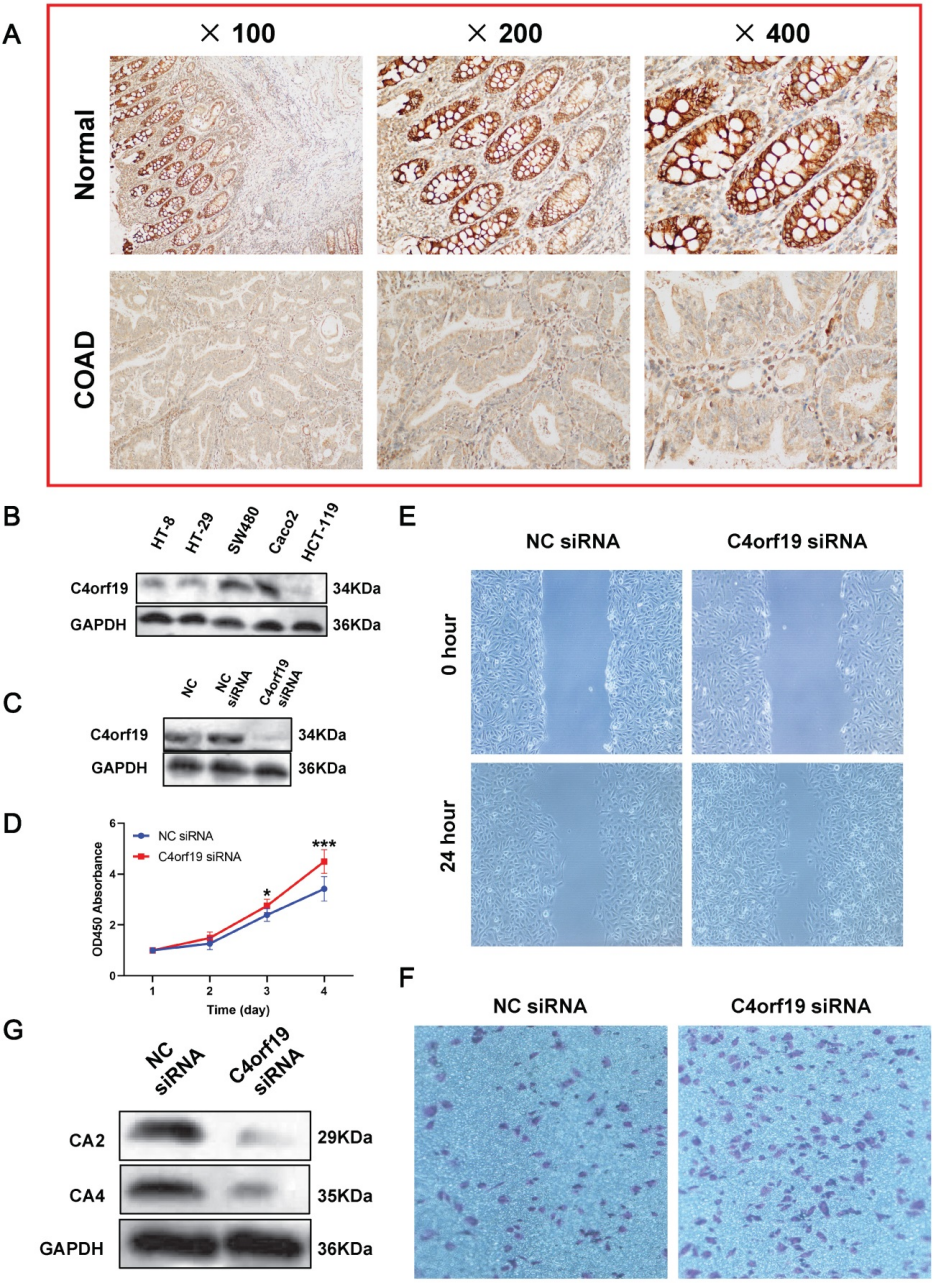
Expression and role of C4orf19 in colorectal adenocarcinoma (COAD). (A) Representative images of positive staining of C4orf19 (marked by red arrows) in normal tissues (upper) and negative staining of C4orf19 in cancer tissues (lower). (B) The expression of C4orf19 in colorectal cancer cells tested by western blot. (C) The expression of C4orf19 protein after transfecting with siRNA for 48h; (D) Effects of C4orf19 siRNA on cell proliferation, *P < 0.05, * *P* < 0.001. (E, F) Effects of C4orf19 siRNA on cell migration (E) and invasion (F). (G) Western blot analysis was performed for CA2 and CA4 expression in caco2 cells after transfection with C4orf19 siRNA or negative control.

First of all, we confirmed that C4orf19 protein expression decreased in COAD tissues by immunohistochemistry **(Figure 8A)**. Therefore, *in vitro* experiments continued to validate the function of C4orf19 in modulating the proliferation, migration and invasion of COAD cells. First, multiple COAD cell lines have been used to detect C4orf19 protein expression. The result indicated that in **Figure 8B**, the protein expression of C4orf19 in Caco2 cells was higher than in other cells. Next, in order to explore the effect of low-regulation C4orf19, we modified the expression of C4orf19 in COAD Caco2 cell lines using a small interfering RNA (siRNA) **(Figure 8C)**. We then observed that the descending expression of C4orf19 favored the rate of caco2 cell growth in comparison with the negative control **(Figure 8D)** compared with the negative control by both CCK8. In the meantime, the transfection of C4orf19 siRNA significantly contributed to the migration (16.21±1.71 vs. 8.30±1.63, *P* < 0.05) and invasion (148±4.55 vs. 86.67±4.64, *P* < 0.001) of the caco2 cell **(Figure 8E and F)**. Beyond this, protein expression levels of CA2 and CA4 were observed dramatically low-regulated in caco-2 cells after transfection with C4orf19 siRNA for 48 hours by western blot **(Figure 8G)**. These results suggested that C4orf19 plays an important role in CRC to partially control the nitrogen metabolic pathway.

## Discussion

As a global health issue, CRC is a highly invasive malignancy with early lymphatic and haematogenic metastases 28. Approximately half of CRC patients die of a recurrent metastatic disease or complications. In this study, we conducted an extensive study to explore CRC's potential mechanism for development and advancement, in order to provide a novel biomarker to predict the prognosis of colon cancer and enhance understanding of CRC's molecular mechanisms.

First, a total of 379 COAD samples and 39 controls were used to detect COAD DEGss. Consequently, 2119 GSE were obtained, of these, 919 were primarily enriched in cell proliferation and cell cycle, and 1200 were primarily related to immune response. In-depth studies have critically considered the role of the cell cycle in the development and development of colorectal cancer 29. At the same time, the immune response plays a crucial role in controlling the temporal progression of colorectal cancer. A close relationship between tumor infiltration rate of lymphocytes and prognosis was found 30. Then, in order to explore the relationship between prognostic genes, WGCNA was used to analyse gene expression data after the Kaplan-Meier estimate. In consequence, 9 modules of prognostic gene co-expression were obtained **(Figure 3)**. Among these nine modules, the brown and pink modules attracted our attention, that have been significantly correlated negatively and positively with all clinical endpoints, including clinical stage, stage T, stage M and stage N, respectively. In addition, the GO and KEGG enrichment analysis showed that the brown module is mainly related to o-glycan processing, carbon metabolism, etc. Several studies have shown that aberrant glycosylation is associated with the development and progression of cancer 31. And some of these glycan changes such as CA 19-9 carbohydrate antigen or those found on carcinoembryonic antigen (CEA) are already used as clinical biomarkers to monitor CRC. In the meantime, the role of carbon metabolism (1CM), in particular folate, in the development of CRC has been extensively studied 32. At the same time, enrichment analysis showed that the pink module genes are primarily associated with vasculogenesis, hypoxia response, etc. Angiogenesis is crucial for the growth, proliferation and metastasization of colorectal cancer 33, whereas studies have shown that hypoxia cancer cells have increased metastatic capability within CRC 34. All of the above analyses imply that the gene from these two modules can play a significant role in carcinogenesis, and gene dysregulation is closely associated with the progression of CRC.

In order to further narrow the field and detect suitable biomarkers, univariate Cox analyses were conducted to detect the core genes of the brown and pink modules. As a result, a total of six protein coding DEGs were obtained, including TEX11, C4orf19, CLCA4, CLCA1, CA2 and NOTCH3. Some of these have been identified as associated with CRC or many other types of cancer. For instance, the expression of TEX11 and NOTCH3 showed a strong correlation with COAD in our work, whereas Luo et al. has shown that TEX11, CDC42, QKI, CAV1 and FN1 were the core genes of early CRC and have potential as biomarkers 35. It was also discovered that NOTCH3, one of the proto-oncogenes, was over-expressed in the CRC. Varga et al. confirmed that the overexpression of NOTCH3 enhanced tumor invasion and metastasis, and positively correlated with tumor grading, lymph node metastasis and distant metastasis, suggesting that NOTCH3 was a therapeutic target for CRC 36.

However, very little is known about the relationship between C4orf19 and cancer. Only Lang and colleagues showed that the expression of C4orf19 was significantly underregulated and could be used as a marker for multifocal and multi-centre breast cancer (MMBC) 37. In this work, we demonstrated that the clinical stage and stage TNM of COAD patients were highly correlated with the expression level of C4orf19. In addition, expression levels of C4orf19 were significantly low-regulated in COAD patients with metastatic lymph nodes or distant metastases. All of these findings suggested that the expression level of C4orf19 could be used as a biomarker not only for the onset of colorectal cancers, but also for the development of the COAD.

In order to further study the potential biological effect of C4orf19 in the COAD, a GSEA analysis was conducted. As can be seen in **Figure 7**, a strong expression of C4orf19 was involved in the nitrogen metabolic pathway. Previous reports have shown that the nitrogen metabolic pathway is closely related to cellular survival, and studies have reported that nitrogen metabolism is extensively involved in malignant tumour progression 38, 39. At the same time, nitric oxide is one of the important mechanisms involved in the initiation, invasion and progression of tumor cells, and epidemiological data suggest that NO has a potential role in cancer development 40. Kodama et al. indicates that carbon and nitrogen metabolism is impaired through the development of malignant tumors. Among these, glutamine nitrogen plays an important role, suggesting that regulating nitrogen metabolism is a potential treatment method for malignant tumors. 26. As in this study, Yu et al. obtained 405 common DEG from GSE9348, GSE22598 and GSE113513 from the GEO database, and through KEGG analysis revealed that ECGs were concentrated in the pathway of nitrogen metabolism, mineral absorption and so forth in colorectal cancer 41.

To explore the role of C4orf19 in CRC and to initially demonstrate the relationship between the expression of C4orf19 and the nitrogen metabolic pathway, *in vitro* studies have been conducted. After C4orf19 silencing, the expressions of CA2 and CA4 were significantly down-regulated, and the invasion ability of caco2 cells was significantly increased, suggesting that C4orf19 played a role in tumor inhibition through regulating the expression of CA2 and CA4 in COAD (**Figure 8**). Mori et al. found that the expression of Carbonic anhydrase in colorectal cancer was significantly higher than that in adjacent normal tissues, and it was positively correlated with the prognosis of colorectal cancer 42. Similarly, Zhang et al. confirmed that CA2 inhibited colorectal cancer proliferation and blockages its cell cycle by inhibiting the Wnt signaling pathway 43.

In conclusion, our work has demonstrated that C4orf19 plays a role in the emergence and development of COAD by regulating nitrogen metabolism. This study provides a potential new marker for the clinical diagnostic of COAD and provides a potential target for the treatment of COAD.

## Supplementary Material

Supplementary figures and tables.
