# Supplementary material for: Down-regulated C4orf19 confers poor prognosis in colon adenocarcinoma identified by gene co-expression network
Source: J Cancer. 2022 Jan 9;13(4):1145–59. doi: 10.7150/jca.63635 (PMC8899380; doi:10.7150/jca.63635)
Supplement: Supplementary file 1 — Supplementary figures and tables. [file jcav13p1145s1.pdf]

Supplementary materials

Supplementary Table S1. Top 50 upregulated and 50 downregulated genes in colon adenocarcinoma (COAD) patients based on TCGA dataset.

Supplementary Table S2. Gene ontology (GO) biological process (BP) term of differentially expressed genes (DEGs) enriched in.

Supplementary Table S3. Gene ontology (GO) biological process (BP) term of down-regulated differentially expressed genes (DEGs).

Supplementary Table S4. Overview of 9 modules constructed by Weighted gene correlation network analysis (WGCNA). (DEG: the differentially expressed genes (DEGs) through the above analysis; COX: prognosis-related genes with $P_{Cox} < 0.05$ explored by univariate Cox analyses. HUB: the 10% of genes with the highest connectivity was defined as hub genes in each module; DEG-COX-HUB: the intersection of DEGs, COX genes and hub genes.)

Supplementary Table S5 The gene sets significantly enriched with high levels of C4orf19 (FDR < 0.05).

Supplementary Figure. S1. The flowchart for obtaining the gene co-expression network and experimental validation

Supplementary Figure. S2. Expression of six hub genes between normal tissues and COAD. *** P < 0.001.

Supplementary Figure. S3. The gene sets significantly enriched with high levels of C4orf19 (FDR < 0.05), ordered by enrichment score.

Supplementary Figure. S4. The gene set of “nitrogen metabolism pathway” was enriched in high levels of C4orf19 (FDR < 0.05). (A) Transcriptional expression profiles of the significant genes were shown in a heat map. (B) GSEA details of nitrogen metabolism pathway.

Supplementary Table S1. Top 50 upregulated and 50 downregulated genes in colon adenocarcinoma (COAD) patients based on TCGA dataset.

upregulated genes				downregulated genes			
Gene	logFC	AveExpr	adj.P.Val	Gene	logFC	AveExpr	adj.P.Val
DPEP1	4.479	5.264	4.22E-29	AQP8	-6.944	2.282	8.88E-74
ETV4	4.215	4.606	1.30E-98	CA1	-6.496	1.551	3.55E-97
FOXQ1	4.093	4.111	9.50E-64	GUCA2B	-6.098	1.648	2.20E-91
CLDN2	3.999	4.243	1.10E-25	ZG16	-6.097	2.801	7.15E-46
CDH3	3.734	3.685	1.73E-98	CLCA4	-6.064	2.231	7.07E-60
CLDN1	3.724	4.304	2.16E-69	GUCA2A	-6.047	4.197	3.00E-53
CST1	3.692	3.474	2.39E-26	CD177	-5.862	1.968	1.54E-71
MMP7	3.625	3.504	3.09E-25	SLC26A3	-5.791	4.563	3.12E-37
CEMIP	3.487	3.744	4.46E-56	TMIGD1	-5.640	1.272	6.51E-111
ASCL2	3.321	5.322	5.74E-32	MS4A12	-5.561	2.012	1.75E-60
KRT23	3.236	3.144	5.48E-18	CA4	-5.465	2.237	1.30E-56
TESC	3.205	4.052	1.42E-30	IGHA2	-5.314	7.468	5.00E-23
MMP1	3.195	4.368	1.80E-20	CA2	-5.194	4.293	3.20E-54
TRIB3	3.195	4.285	1.88E-67	CEACAM7	-5.103	5.071	7.80E-34
KRT80	3.116	2.934	5.99E-63	CLCA1	-5.092	4.176	2.15E-23
GDF15	3.064	5.973	2.05E-42	CHGA	-5.008	1.761	1.36E-65
MMP11	2.981	3.781	3.90E-31	JCHAIN	-4.999	6.450	9.59E-27
LARGE2	2.974	3.366	1.88E-42	PYY	-4.947	1.164	2.82E-94
TGFBI	2.947	6.411	4.11E-42	CA7	-4.947	1.200	1.10E-138
C6orf223	2.944	3.005	2.75E-52	ADH1C	-4.718	3.383	2.70E-44
SLC7A5	2.941	5.113	3.78E-63	OTOP2	-4.629	0.563	4.06E-202
CPNE7	2.929	2.917	6.84E-37	ANPEP	-4.561	3.600	1.85E-43
MMP3	2.925	3.293	7.63E-20	BEST4	-4.430	1.120	7.06E-154
CA9	2.854	3.231	9.14E-16	SLC26A2	-4.338	3.409	4.26E-48
SLCO4A1	2.834	3.964	3.01E-58	CHP2	-4.301	3.129	2.82E-44
S100P	2.825	6.906	1.38E-28	LYPD8	-4.209	3.291	1.10E-36
CXCL8	2.811	4.063	2.22E-19	MT1M	-4.156	1.835	4.36E-61
TNS4	2.746	4.250	8.36E-32	DES	-4.151	4.212	2.11E-21
PRSS22	2.741	3.084	1.71E-46	SLC4A4	-4.039	1.311	5.92E-90
NOTUM	2.739	2.535	6.35E-12	MYH11	-4.019	2.986	2.18E-35
ELFN1-AS1	2.679	2.776	2.68E-35	ITLN1	-4.018	3.682	3.26E-18
CXCL1	2.679	5.239	1.21E-24	UGT2B17	-4.017	2.935	4.78E-21
GRIN2D	2.663	2.758	2.70E-49	IGHA1	-3.962	8.658	6.86E-17
LY6G6D	2.626	2.701	2.62E-12	SLC51B	-3.916	2.596	4.80E-62
SCD	2.619	6.214	3.05E-39	FCGBP	-3.861	4.438	7.05E-22

PDX1	2.605	2.697	9.94E-44	MT1H	-3.695	2.119	7.71E-48
SPP1	2.539	4.339	4.16E-12	MT1G	-3.687	4.988	1.55E-32
TNFRSF12A	2.526	4.752	7.54E-59	FABP1	-3.686	6.503	3.15E-20
AZGP1	2.523	4.296	1.03E-24	SCARA5	-3.653	1.068	1.78E-117
CTHRC1	2.521	3.424	5.58E-26	CLEC3B	-3.633	1.840	4.06E-107
NFE2L3	2.516	4.812	1.28E-73	VSIG2	-3.630	3.407	1.14E-24
KLK6	2.504	2.298	2.76E-14	SST	-3.615	0.773	4.18E-101
TRIM29	2.453	2.689	2.54E-28	PKIB	-3.613	1.922	8.64E-71
CXCL3	2.435	3.862	8.53E-25	SCNN1B	-3.611	1.463	2.18E-67
CEL	2.432	2.486	1.80E-11	IGHV3-74	-3.565	3.925	4.08E-22
FABP6	2.411	2.728	8.34E-21	ADAMDEC1	-3.561	3.364	4.64E-46
NKD2	2.404	2.697	1.65E-25	NXPE4	-3.548	3.059	1.21E-24
PSAT1	2.401	4.491	7.92E-36	ADH1B	-3.544	0.947	1.54E-85
SERPINB5	2.366	3.140	1.12E-15	IGLV2-8	-3.532	3.655	1.21E-22
SLC6A6	2.326	3.632	3.40E-40	AKR1B10	-3.529	2.950	1.73E-35

Supplementary Table S2. Gene ontology (GO) biological process (BP) term of up-regulated differentially expressed genes (DEGs).

GO NO.	BP Term	Count	GeneRatio(%)	FDR
GO:0000082	G1/S transition of mitotic cell cycle	27	3.195	1.93E-10
GO:0051301	cell division	50	5.917	1.93E-10
GO:0007067	mitotic nuclear division	38	4.497	2.08E-08
GO:0006260	DNA replication	29	3.432	4.76E-08
GO:0030574	collagen catabolic process	18	2.130	4.62E-07
GO:0006364	rRNA processing	31	3.669	3.74E-06
GO:0008283	cell proliferation	41	4.852	1.60E-05
GO:0006865	amino acid transport	12	1.420	4.38E-05
GO:0007062	sister chromatid cohesion	19	2.249	9.32E-05
GO:0000281	mitotic cytokinesis	10	1.183	5.73E-04
GO:0008284	positive regulation of cell proliferation	43	5.089	8.88E-04
GO:0030199	collagen fibril organization	11	1.302	8.88E-04
GO:0006270	DNA replication initiation	10	1.183	1.10E-03
GO:0042127	regulation of cell proliferation	23	2.722	2.51E-03
GO:0031100	organ regeneration	11	1.302	4.26E-03
GO:0007059	chromosome segregation	13	1.538	4.57E-03
GO:0006268	DNA unwinding involved in DNA replication	6	0.710	4.96E-03
GO:0000070	mitotic sister chromatid segregation	8	0.947	9.61E-03
GO:0000086	G2/M transition of mitotic cell cycle	18	2.130	1.04E-02
GO:0048146	positive regulation of fibroblast proliferation	11	1.302	1.13E-02
GO:0030198	extracellular matrix organization	22	2.604	1.24E-02
GO:0034475	U4 snRNA 3'-end processing	5	0.592	2.64E-02
GO:0006977	DNA damage response, signal transduction by p53 class mediator resulting in cell cycle arrest	11	1.302	3.26E-02
GO:0006271	DNA strand elongation involved in DNA replication	6	0.710	3.26E-02
GO:1904874	positive regulation of telomerase RNA localization to Cajal body	6	0.710	3.26E-02
GO:0006564	L-serine biosynthetic process	4	0.473	3.26E-02
GO:0000083	regulation of transcription involved in G1/S transition of mitotic cell cycle	7	0.828	3.26E-02

GO:0032212	positive regulation of telomere maintenance via telomerase	8	0.947	3.26E-02
GO:0022617	extracellular matrix disassembly	12	1.420	3.26E-02
GO:0030324	lung development	12	1.420	3.26E-02
GO:0034427	nuclear-transcribed mRNA catabolic process, exonucleolytic, 3'-5'	5	0.592	3.26E-02
GO:0007051	spindle organization	6	0.710	3.70E-02
GO:0001649	osteoblast differentiation	14	1.657	3.94E-02
GO:0031145	anaphase-promoting complex-dependent catabolic process	12	1.420	4.12E-02
GO:0098609	cell-cell adhesion	25	2.959	4.49E-02

Supplementary Table S3. Gene ontology (GO) biological process (BP) term of down-regulated differentially expressed genes (DEGs).

GO NO.	BP Term	Count	GeneRatio(%)	FDR
GO:0006958	complement activation, classical pathway	61	5.365	1.54E-47
GO:0006956	complement activation	55	4.837	1.24E-43
GO:0006898	receptor-mediated endocytosis	68	5.981	4.55E-35
GO:0006955	immune response	91	8.004	6.70E-28
GO:0038096	Fc-gamma receptor signaling pathway involved in phagocytosis	49	4.310	6.92E-26
GO:0050776	regulation of immune response	55	4.837	6.28E-24
GO:0038095	Fc-epsilon receptor signaling pathway	50	4.398	1.40E-19
GO:0006508	proteolysis	82	7.212	4.33E-17
GO:0050853	B cell receptor signaling pathway	19	1.671	5.92E-08
GO:0006910	phagocytosis, recognition	14	1.231	1.51E-07
GO:0006911	phagocytosis, engulfment	14	1.231	3.82E-06
GO:0050871	positive regulation of B cell activation	12	1.055	9.44E-06
GO:0006936	muscle contraction	22	1.935	4.66E-05
GO:0071456	cellular response to hypoxia	19	1.671	6.43E-04
GO:0070374	positive regulation of ERK1 and ERK2 cascade	26	2.287	1.43E-03
GO:0006874	cellular calcium ion homeostasis	17	1.495	6.00E-03
GO:0045926	negative regulation of growth	8	0.704	6.00E-03
GO:0071294	cellular response to zinc ion	8	0.704	6.00E-03
GO:0071377	cellular response to glucagon stimulus	11	0.967	6.00E-03
GO:0002548	monocyte chemotaxis	11	0.967	8.98E-03
GO:0007166	cell surface receptor signaling pathway	32	2.814	9.92E-03
GO:0006954	inflammatory response	40	3.518	9.92E-03
GO:0015701	bicarbonate transport	11	0.967	1.16E-02
GO:0070098	chemokine-mediated signaling pathway	14	1.231	1.16E-02
GO:0002250	adaptive immune response	21	1.847	1.54E-02
GO:0071276	cellular response to cadmium ion	7	0.616	2.13E-02
GO:0071346	cellular response to interferon-gamma	12	1.055	2.19E-02
GO:0006805	xenobiotic metabolic process	14	1.231	2.66E-02

Supplementary Table S4. Overview of 9 modules constructed by Weighted gene correlation network analysis (WGCNA). (DEG: the differentially expressed genes (DEGs) through the above analysis; COX: prognosis-related genes with $P_{Cox} < 0.05$ explored by univariate Cox analyses.HUB: the 10% of genes with the highest connectivity was defined as hub genes in each modules; DEG-COX-HUB: the intersection of DEGs, COX genes and hub genes.)

**Module Name**	**Size (n)**	**DEGs (n)**	**COX (n)**	**DEG-COX-HUB**	**Correlation with important clinic parameters**
turquoise	749	1	110	-	-
blue	231	1	104	-	COAD: r = 0.19 P = 7.4E-05
brown	228	95	129	AP001554.1 TEX11 C4orf19 CLCA4 AC016027.1 CLCA1 CA2	COAD: r = - 0.82 P = 2.4E-102<br>Clinical Stage: r = - 0.2 P = 4.7E-05<br>T stage: r = -0.19 P = 0.00012<br>M stage: r = -0.17 P = 0.00034<br>N stage: r = -0.2 P = 3.2E-05
yellow	222	42	61	SPARCL1 CADM3 CRYAB ArHGEF25 CHRDL1 PRELP	COAD: r = - 0.56 P = 4.0E-36<br>Clinical Stage: r = 0.19 P = 9.8E-05<br>T stage: r = 0.17 P = 0.00047<br>M stage: r = 0.12 P = 0.018<br>N stage: r = 0.24 P = 5.3E-07
green	210	13	166	-	-
red	192	0	35	-	Clinical Stage: r = 0.11 P = 0.026<br>T stage: r = 0.11 P = 0.019<br>M stage: r = 0.12 P = 0.011<br>N stage: r = 0.14 P = 0.0054
black	158	0	87	-	N stage r = 0.14 P = 0.0036
pink	116	1	34	NOTCH3	COAD: r = 0.16 P = 0.0012<br>Clinical Stage: r = 0.16 P = 0.00073<br>T stage: r = 0.19 P = 0.00011<br>N stage: r = 0.21 P = 1.4E-05
magenta	48	0	182	-	N stage r = 0.15 P = 0.0027

Supplymentary Table S5 The gene sets significantly enriched with high levels of C4orf19.(FDR < 0.05)

NAME	SIZE	NES	NOM p-val	FDR q-val
**NITROGEN_METABOLISM**	**23**	**2.176**	**0.000**	**0.012**
STARCH_AND_SUCROSE_METABOLISM	52	2.155	0.000	0.010
RETINOL_METABOLISM	64	2.134	0.000	0.010
PORPHYRIN_AND_CHLOROPHYLL_METABOLISM	40	2.111	0.000	0.011
ASCORBATE_AND_ALDARATE_METABOLISM	25	2.020	0.004	0.024
PENTOSE_AND_GLUCURONATE_INTERCONVERSIONS	28	2.007	0.000	0.025
AMINO_SUGAR_AND_NUCLEOTIDE_SUGAR_METABOLISM	43	2.026	0.000	0.025
VALINE_LEUCINE_AND_ISOLEUCINE_DEGRADATION	43	1.970	0.006	0.030
GLYCOSPHINGOLIPID_BIOSYNTHESIS_LACTO_AND_NEOLACTO_SERIES	26	1.944	0.000	0.031
DRUG_METABOLISM_CYTOCHROME_P450	71	1.961	0.000	0.031
ONE_CARBON_POOL_BY_FOLATE	17	1.922	0.010	0.032
CITRATE_CYCLE_TCA_CYCLE	31	1.926	0.008	0.033
DRUG_METABOLISM_OTHER_ENZYMES	51	1.978	0.000	0.033
TERPENOID_BACKBONE_BIOSYNTHESIS	15	1.945	0.004	0.034
BUTANOATE_METABOLISM	34	1.928	0.008	0.034
METABOLISM_OF_XENOBIOTICS_BY_CYTOCHROME_P450	69	1.905	0.002	0.037
PEROXISOME	78	1.896	0.010	0.038
FATTY_ACID_METABOLISM	42	1.860	0.009	0.050

Supplementary Figure. S1. The flowchart for obtaining the gene co-expression network and experimental validation

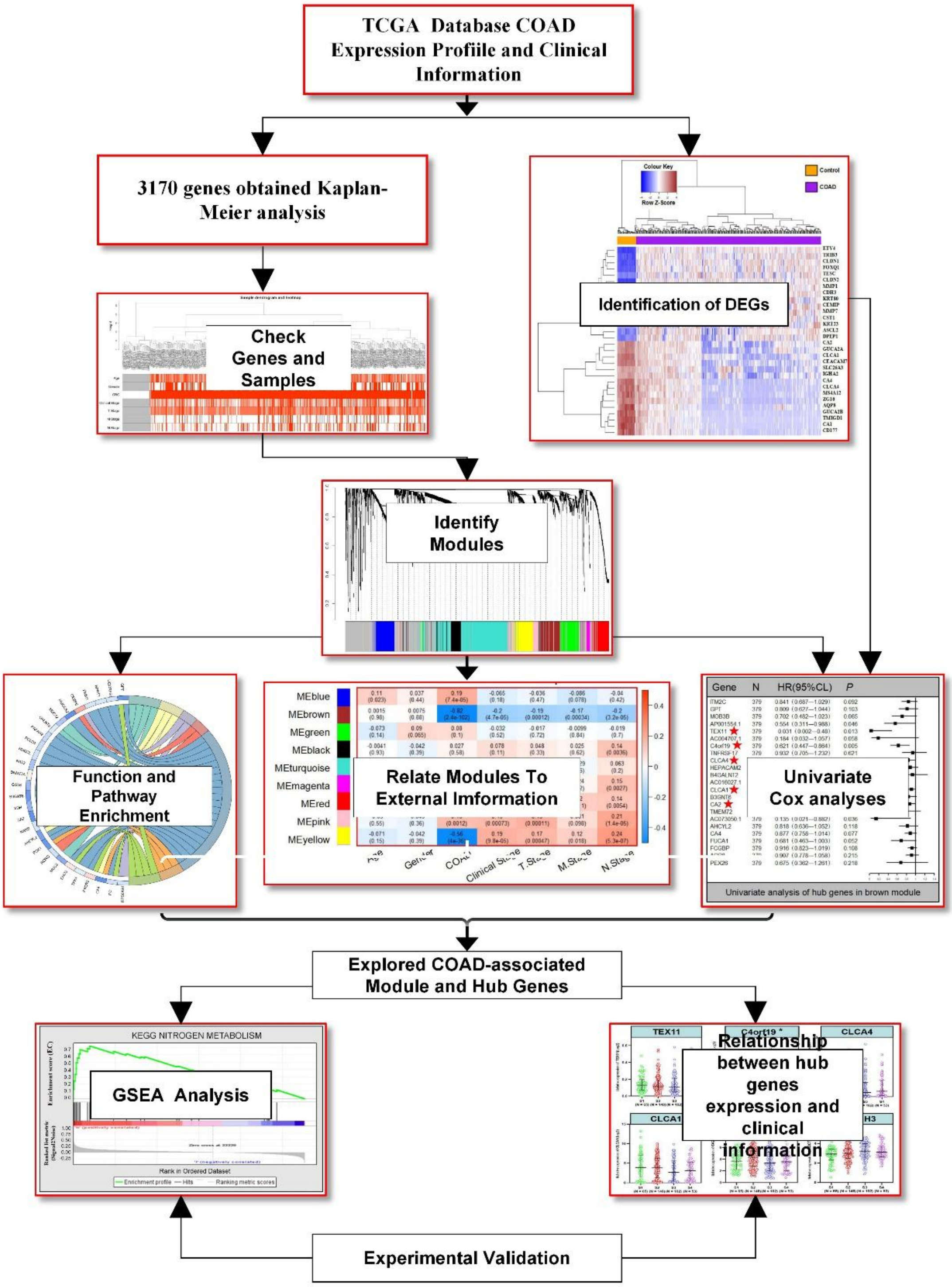

Supplementary Figure. S2. Expression of six hub genes between normal tissues and COAD. *** P < 0.001.

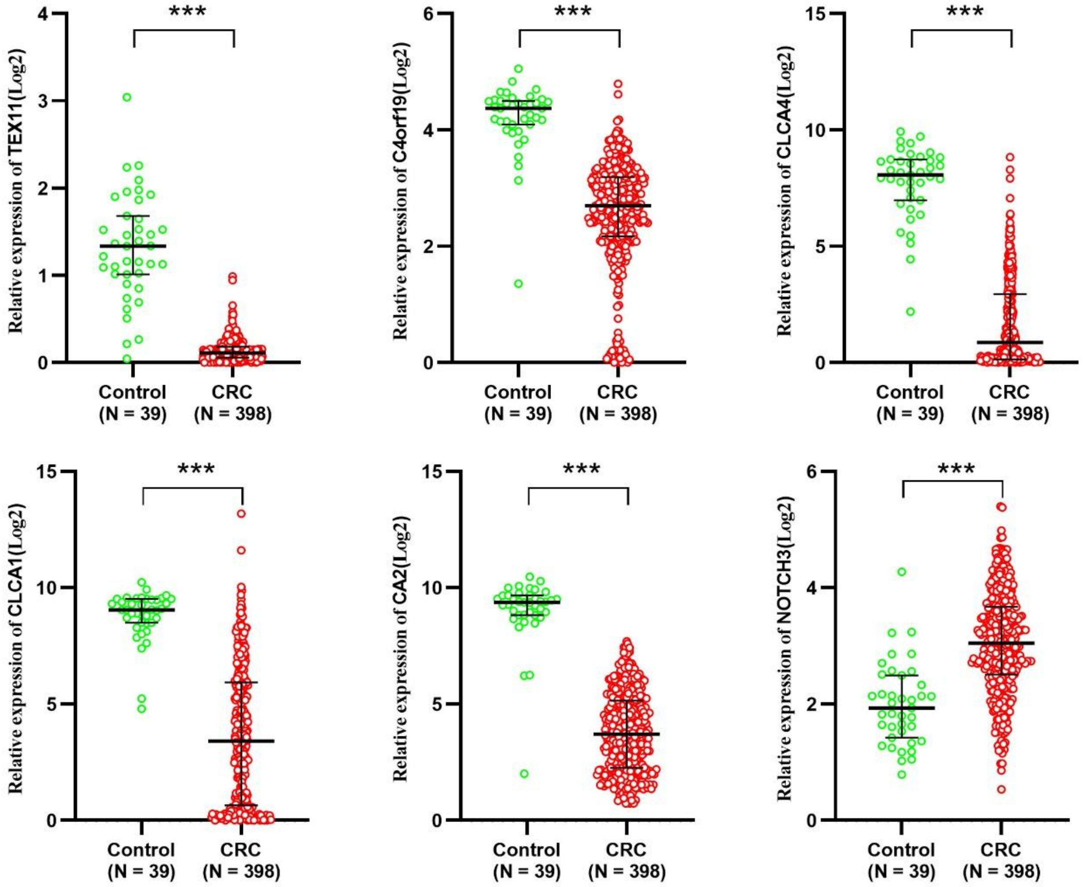

Supplementary Figure. S3. The gene sets significantly enriched with high levels of C4orf19 (FDR < 0.05), ordered by enrichment score.

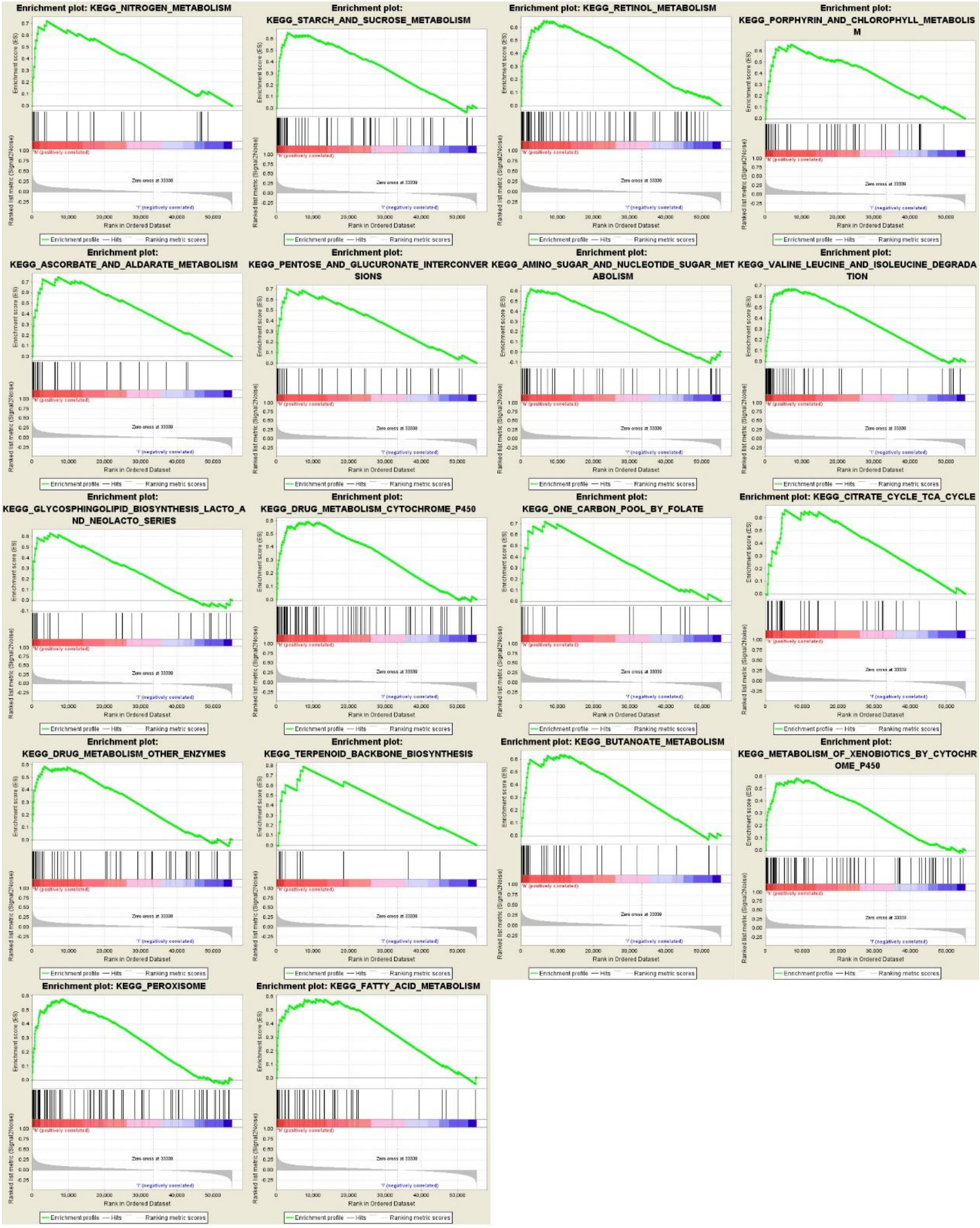

Supplementary Figure. S4. The gene set of “nitrogen metabolism pathway” was enriched in high levels of C4orf19 (FDR < 0.05). (A) Transcriptional expression profiles of the significant genes were shown in a heat map. (B) GSEA details of nitrogen metabolism pathway.

A

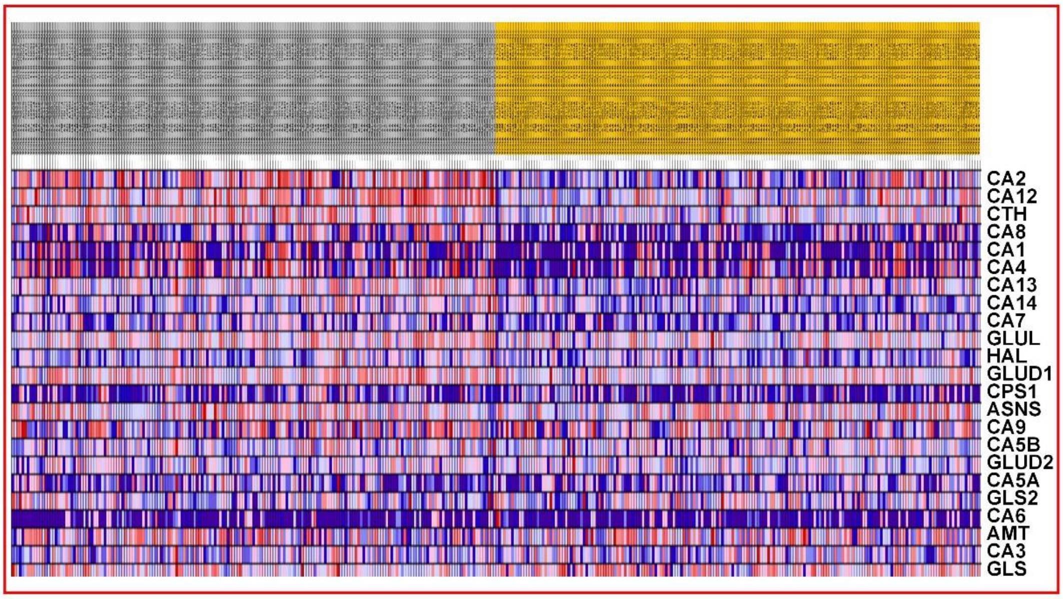

B

	PROBE	DESCRIPTION (from dataset)	GENE SYMBOL	GENE_TITLE	RANK IN GENE LIST	RANK METRIC SCORE	RUNNING ES	CORE ENRICHMENT
1	CA2	na			80	0.394	0.1258	Yes
2	CA12	na			124	0.368	0.2440	Yes
3	CTH	na			368	0.295	0.3351	Yes
4	CA8	na			640	0.253	0.4120	Yes
5	CA1	na			703	0.246	0.4905	Yes
6	CA4	na			940	0.224	0.5586	Yes
7	CA13	na			1360	0.200	0.6157	Yes
8	CA14	na			1662	0.189	0.6712	Yes
9	CA7	na			3365	0.144	0.6870	Yes
10	GLUL	na			3868	0.136	0.7218	Yes
11	HAL	na			9562	0.087	0.6470	No
12	GLUD1	na			12645	0.073	0.6147	No
13	CPS1	na			16033	0.060	0.5727	No
14	ASNS	na			17043	0.056	0.5725	No
15	CA9	na			24751	0.028	0.4422	No
16	CA5B	na			25214	0.027	0.4425	No
17	GLUD2	na			28217	0.016	0.3934	No
18	CA5A	na			30022	0.010	0.3641	No
19	GLS2	na			45661	-0.049	0.0967	No
20	CA6	na			46304	-0.053	0.1022	No
21	AMT	na			46631	-0.056	0.1143	No
22	CA3	na			46937	-0.058	0.1275	No
23	GLS	na			48590	-0.072	0.1208	No
